# Risk factors of stillbirth in rural China: A national cohort study

**DOI:** 10.1038/s41598-018-35931-1

**Published:** 2019-01-23

**Authors:** Yimin Qu, Shi Chen, Hui Pan, Huijuan Zhu, Chengsheng Yan, Shikun Zhang, Yu Jiang

**Affiliations:** 1 School of Public Health, Chinese Academy of Medical Sciences & Peking Union Medical College (CAMS & PUMC), Beijing, 100730 China; 2 Department of Endocrinology, Key Lab of Endocrinology, Ministry of Health,Peking Union Medical College Hospital (PUMCH), Chinese Academy of Medical Sciences & Peking Union Medical College (CAMS & PUMC), Beijing, 100730 China; 3 Hebei Center for women and children’s health, Shijiazhuang, 050031 China; 4 Research association for women and children’s health, Beijing, 100081 China; 5 The Jockey Club School of Public Health and Primary Care, The Chinese University of Hong Kong, Hong Kong, China

## Abstract

People living in rural China are more frequently exposed to some specific risk factors which made stillbirth rate higher than urban areas. National Free Preconception Health Examination Project was launched to investigate these risk factors and collected a representative sample of 248501 participants from 31 provinces in China from 2010 to 2013. Parental risk factors were ascertained twice before and during pregnancy respectively by questionnaires. Stillbirth or live birth were recorded by trained physicians. In the analysis, nested case-control study was conducted, and propensity score matching method was used to adjust the confounding. Multi-level logistic regression was used to fit for multi-level sampling. The overall stillbirth rate was 0.35% in rural China, it was higher in North (0.42%) and West (0.64%) areas. Maternal exposure to pesticide (OR (95%CI 1.06, 3.39)), hypertension (OR = 1.58 (95%CI 1.07, 2.34)), lack of appetite for vegetables (OR = 1.99 (95%CI 1.00, 3.93)), stress (compared with no pressure, OR of a little pressure was 1.34(95% CI 1.02, 1.76)); paternal exposure to smoking (OR = 1.22 (95% CI 1.02, 1.46)), organic solvents (OR = 1.64 (95% CI 1.01, 2.69)) were found independent risk factors of stillbirth. Folacin intake 3 months before pregnancy (OR = 0.72 (95%) CI 0.59, 0.89), folacin intake 1-2 months before pregnancy (OR = 0.71 (95% CI 0.55, 0.92)), folacin intake after pregnancy (OR = 0.81 (95% CI 0.65, 1.02) for) were protect factors of stillbirth. Maternal pesticide exposure, lack of vegetables, stress, paternal smoking and exposure to organic solvents were risk factors of stillbirth. Folic acid intake was protective factor of stillbirth, no matter when the intake began.

## Introduction

Women and children’s health is a priority of public health in China now. The stillbirth rate in China has declined by 4–6% from 2000 to 2015, but the newborn survival has lagged behind maternal and under-five survival the rate, the stillbirth rate declined more slowly than other adverse maternal and infant outcomes^1^. To end preventable stillbirths, the Every Newborn Action Plan, a global multi-partner movement set a target for national stillbirth rates of 12 or fewer stillbirths per 1000 births in all countries by 2030 in 2014. Disparities within countries should also be addresses^2,3^. As an agricultural country, 43.9% of Chinese total population live in rural areas^4^. Compared with city women, women in rural China do face inequalities in health services and access to health education and promotion, and are generally more exposed to risk factors such as pesticide, unhealthy diet, and inadequate folate intake due to limited resources available, economical underdevelopment, and substandard education level^5–9^. Besides, the living and environmental condition are also worse in rural areas^10^.

Access to health services, parental health-related behaviors such as smoking, drinking, physical activities and folacin intake are established to be risk factors of many adverse pregnancy outcomes^11–15^. No national perspective investigation about the epidemic of these risk factors and their relationship with stillbirth in rural China was conducted before.

To get a better understanding of maternal and newborn health of rural China, and identify current risk factors so as to improve maternal and newborn health, the government launched the “National Free Preconception Health Examination Project” in rural China from January 2010. Our study was based on the data on couples enrolled during 2010–12, which is the first set of the project data^16,17^.

## Study Design

### Participants

A two-stage stratified cluster sampling method was used for the recruitment of participants aged 21∼49, planning to deliver a baby within 6 months from 220 counties of 31 provinces, 86% of the target population was covered^18^. For all 31 provinces, counties were selected in the same proportion based on the population size and numbers of counties in each province^17^. All eligible couples living in these selected counties have access to this project, and 86% of target population were covered^18,19^.

A standardized questionnaire about the general information before the pregnancy including parental basic characteristics, childbearing history, living habit, and other exposing status before and during pregnancy were collected at the time of enrollment to the project by the local health workers^20^. Then they were followed up by telephone interview every 2–3 months to determine the conception status. Women who became pregnant within 6 months were closely followed up, and another standardized family heath file about health exposures during the pregnancy and medical examination data was recorded. Pregnancy outcomes were collected according to hospital health record within 6 weeks after delivery or 2 weeks within other pregnancy outcome by doctors. For those participants of whom no relevant hospital health record can be found, telephone follow-up was conducted. All records are uploaded in a web-based electronic data collection system^16,17,20^.

By December 2012, 248501 families achieved the whole follow-up of this project, and their pregnancy outcomes were recorded by doctors. Pregnancy outcomes including birth defects and multiple gestations were excluded from the analysis considering the undiagnosed genetic conditions.

### Definition and Assessment on variables

Stillbirth was defined as fetal death on or after 20 weeks of gestation in this study. Maternal BMI in adults was categorized into four groups: <18.5, 18.5–23.9; between 24.0–27.9, ≥28.0 kg/m^21^. Advanced maternal age was defined as first pregnancy on or after the age of 35. Past medical history of hypertension or systolic blood pressure >140 or diastolic blood pressure >90 were considered as high blood pressure. Parental education levels were classified into illiteracy, primary school, junior middle school, senior middle school, undergraduate, postgraduate and above according to the current education system. Parental occupation was classified into farmer, physical worker, service industry laborer, businessman, house worker, office clerk, and others.

We divided the participants geographically according to the Qinling-Huaihe line, and Heihe-Tengchong line. Qinling-Huaihe line bisects China into north and south regions, culture, climate, living habits are all different between north and south, which affect people’s health situation^22^. Heihe-Tengchong line divides China from Heihe in Heilongjiang province to Tengchong in Yunnan province into east and west. The east is more economically developed with higher civilization degree than west, and it takes up 96% of total Chinese population even though it only covers 36% areas of China. The economic imbalance between east and west resulted the huge differences in health resources^23^.

Environmental exposure as pesticide and new decoration were classified into two groups, no and yes, social pressure was classified to five levels, with 1 the lowest and 5 the highest. Folacin intake duration was classified into more than 3 months before pregnancy, 1–2 months before pregnancy, after pregnancy, and no. And whether they take folacin regularly was also asked.

### Statistical analysis

To control multiple confounding between the cases and controls and address severe imbalance between numbers of case and control which may cause high misclassification of interested outcome by logistic regression^24^, nested case-control study was conducted, and propensity score matching method was used to find 8 controls for each case. Logistic regression was used to calculate the propensity score, confounding factors as area^22,23^, breeding history^25,26^, education level^27,28^, occupation^29^, age group^30^ were adjusted in this procedure. Parental age, education level, occupation were closed related and can’t be put into the logistic regression together, we select maternal age, education level^27,28^, and paternal occupation as the dependent variables, as they were commonly used in the scoring of family social economic status, and were reported to be more closed related with the family utilization of health resources and family health status^31^. Wald χ^2^ was used for the model’s overall significance test, Hosmer-Lemeshow was used for the goodness test. After propensity score matching, cases and selected controls were used for further risk factors analysis.

T-test and McNemar test were conducted respectively for continuous data and categorical data in the univariate analysis. Due to differences in climate, culture and economics between different provinces, the subjects may share some homogeneity within a province and heterogeneity between provinces, individually independence required by traditional logistic regression was not matched. As a result, multi-level logistic regression was used to compare the exposures between case and control group. Province was set as the first level and individuals as the second level. Due to collinearity between some exposures between parents, models were built separately for maternal exposures and paternal exposures. Risk factors which were statistically significant in univariate analysis or were proven to be confounders in previous studies were included in the models. GLM procedure in SAS was used to build the model, parameters as Tolerance (TOL) and Variance Inflation Factor (VIF) were used to detect the collinearity between variables^32^. Intra-Class Correlation Coefficient (ICC) was used to test the independence and heterogeneity within level.

All data were expressed as mean ± SD or count (percentages), as appropriate. All statistical procedures were analyzed by SAS 9.4.

## Results

The overall stillbirth rate was 0.35% in rural China, it was higher in north (0.42%) and west (0.64%) than in South (0.32%) and east (0.34%).

Before the propensity score matching, parental education level, occupation, age group, height, weight, were all significantly different between stillbirth group and live birth group (Table 1). Maternal illiteracy group had a stillbirth rate of 0.60%, while mothers with education level equal or higher than postgraduate had a stillbirth rate of 0.64%, both significantly higher than the average rate. Parental advanced age group also had higher stillbirth rate, 0.54% for paternal advanced age and 0.77% for maternal advanced age. Beside, stillbirth group had average lower parental height.

**Table 1:** Comparision of Original Basic Parental characteristics between stillbirth and live birth.

Group	Paternal		*P*	Maternal		*P*
	Live birth N = 229917	Stillbirth N = 811		Live birth N = 229917	Stillbirth N = 811	
Education level			<0.0001*			
illiteracy	232(99.57)	1(0.43)		500(99.40)	3(0.60)	<0.0001*
Primary school	9061(99.40)	55(0.60)		10721(99.38)	67(0.62)	
Junior middle school	139110(99.63)	510(0.37)		145005(99.64)	521(0.36)	
Senior middle school	49847(99.73)	135(0.27)		45774(99.71)	133(0.29)	
undergraduate	26363(99.68)	85(0.32)		24031(99.7)	72(0.30)	
Postgraduate and above	494(99.60)	2(0.40)		311(99.36)	2(0.64)	
Occupation			0.0404*			0.0036*
Farmer	159211(99.62)	607(0.38)		165020(99.62)	627(0.38)	
Physical worker	33247(99.73)	90(0.27)		26349(99.75)	67(0.25)	
service industry	8625(99.68)	28(0.32)		9583(99.81)	18(0.19)	
businessman	8151(99.68)	26(0.32)		4703(99.56)	21(0.44)	
house worker	311(100.00)	0(0.00)		5538(99.66)	19(0.34)	
office clerk	8115(99.68)	26(0.32)		8266(99.61)	32(0.39)	
others	6138(99.74)	16(0.26)		5285(99.68)	17(0.32)	
Age group			<0.0001*			<0.0001*
<35	210902(99.67)	699(0.33)		221395(99.66)	747(0.34)	
≥35	14664(99.46)	80(0.54)		5965(99.23)	46(0.77)	
Age(year)	26.72 ± 4.41	27.43 ± 4.98	<0.0001*	24.60 ± 3.91	25.43 ± 4.64	<0.0001*
height(cm)	171.1 ± 5.12	170.6 ± 6.29	0.0147*	159.2 ± 4.81	158.7 ± 4.99	0.0060*
weight(kg)	65.60 ± 9.12	65.55 ± 9.57	0.8925	53.36 ± 7.26	53.72 ± 7.53	0.1727
BMI(kg/m^2^)	22.39 ± 3.38	22.59 ± 4.19	0.2140	21.08 ± 3.52	21.32 ± 2.68	0.0141*

*For *P* < 0.05.

By propensity score matching, 8 controls were found for each case. Breeding history, living region, maternal education level, maternal age, paternal occupation were taken into consideration in the matching process. The logistic regression used to calculate the propensity score was statistically significant, with*χ*^2^_*Wald*_ = 127.04, *P* < 0.0001, and the Hosmer-Lemeshow test showed that the goodness of fit of the model is good, with*χ*^2^_*HL*_ = 3.56, *P* = 0.8946. After the propensity score matching, basic characteristics were all equally distributed between the two groups (Table 2). Following analysis were conducted with the two groups of 811 cases and 6488 controls.

**Table 2:** Comparison of Basic Parental characteristics after Propensity Score Matching.

Exposure	group	Live birth (n = 6488)	Stillbirth (n = 811)	*P*
Maternal education level	illiteracy	18(0.28)	3(0.38)	0.9628
	Primary school	547(8.50)	67(8.40)	
	Junior middle school	4136(64.23)	521(65.29)	
	Senior middle school	1144(17.77)	133(16.67)	
	undergraduate	582(9.04)	72(9.02)	
	Postgraduate and above	12(0.19)	2(0.25)	
Maternal occupation	Farmer	4934(77.32)	627(78.28)	0.1554
	Physical worker	577(9.04)	67(8.36)	
	service industry	256(4.01)	18(2.25)	
	businessman	116(1.82)	21(2.62)	
	house worker	152(2.38)	19(2.37)	
	office clerk	231(3.62)	32(4.00)	
	others	115(1.80)	17(2.12)	
Paternal education level	illiteracy	9(0.14)	1(0.13)	0.2914
	Primary school	395(6.17)	55(6.98)	
	Junior middle school	3993(62.4)	510(64.72)	
	Senior middle school	1326(20.72)	135(17.13)	
	undergraduate	665(10.39)	85(10.79)	
	Postgraduate and above	11(0.17)	2(0.25)	
Paternal occupation	Farmer	4751(74.82)	607(76.54)	0.9257
	Physical worker	767(12.08)	90(11.35)	
	service industry	261(4.11)	28(3.53)	
	businessman	213(3.35)	26(3.28)	
	house worker	215(3.39)	26(3.28)	
	office clerk	143(2.25)	16(2.02)	
area	north	2294(35.36)	294(36.25)	0.6159
	south	4194(64.64)	517(63.75)	
	east	5948(91.68)	749(92.36)	0.5080
	west	540(8.32)	62(7.64)	
breeding history	0	3536(56.05)	439(55.85)	0.4392
	≥1	2773(43.95)	347(44.15)	
Maternal age		25.45 ± 4.56	25.43 ± 4.64	0.8921
Paternal age		27.46 ± 4.88	27.43 ± 4.98	0.8707

As to parental health behaviors before pregnancy, Mc-Nemar test result showed that maternal passive smoking (P = 0.0443, OR = 1.56 (95%CI 1.01, 2.41)), lack of vegetables (P = 0.0165, OR = 2.25 (95%CI 1.16, 4.39)), exposed to pesticide before pregnancy (P = 0.0047, OR = 2.24 (1.26, 3.97)), hypertension (P = 0.006, OR = 1.73 (95%CI 1.17, 2.56)) were related with stillbirth. Paternal smoking (P = 0.0025, OR = 1.28 (95%CI 1.09, 1.50)), and paternal exposed to new decoration before pregnancy (P = 0.0287, OR = 1.71 (1.05, 2.77)) were related with stillbirth. While maternal smoking (P = 0.3042, OR = 1.65(95% CI 0.63, 4.31)), paternal lack of vegetables (P = 0.4584, OR = 0.36 (95%CI 0.60, 3.08)), paternal passive smoking (P = 0.0978, OR = 1.36 (95%CI 0.95, 1.96)) were not found statistically significantly related with stillbirth in our study (Table 3).

**Table 3:** Univariate analysis of risk factors for stillbirth.

exposure	status	Live birth (n = 6488)	Stillbirth (n = 811)	*P*	OR(95% *CI*)
Lack of vegetables	No(♀)	6242(99.32)	776(98.48)	0.0165	
	Yes(♀)	43(0.68)	12(1.52)		2.25(1.16, 4.39)*
	No(♂)	5924(99.33)	736(99.06)	0.4584	
	Yes(♂)	40(0.67)	7(0.94)		0.36(0.60, 3.08)
Smoking	No(♀)	6249(99.62)	783(99.37)	0.3042	
	Yes(♀)	24(0.38)	5(0.63)		1.65(0.63, 4.31)
	No(♂)	3923(65.83)	446(60.11)	0.0025	
	Yes(♂)	2036(34.17)	296(39.89)		1.28(1.09, 1.50)*
Passive smoking	No or seldom(♀)	6145(97.83)	761(96.70)	0.0443	
	often(♀)	136(2.17)	26(3.30)		1.56(1.01, 2.41)*
	No or seldom(♂)	5742(96.41)	707(95.15)	0.0978	
	often(♂)	214(3.59)	36(4.85)		1.36(0.95, 1.96)
Hypertension	No(♀)	6078(97.44)	733(95.69)	0.006	
	Yes(♀)	160(2.56)	33(4.31)		1.73(1.17, 2.56)*
Pesticide Before pregnancy	No(♀)	6428(99.08)	795(98.03)	0.0047	
	Yes(♀)	60(0.92)	16(1.97)		2.24(1.26, 3.97)*
	No(♀)	6412(98.83)	795(98.03)	0.0516	
	Yes (♂)	76(1.17)	16(1.97)		1.70(0.99, 2.94)
	No (♀)	6420(99.60)	808(99.75)	0.4982	
	Yes (♀)	26(0.40)	2(0.25)		0.62(0.15, 2.58)
New decoration Before pregnancy	No (♀)	6408(98.77)	800(98.64)	0.7614	
	Yes (♀)	80(1.23)	11(1.36)		1.10(0.58, 2.09)
During pregnancy	No (♂)	6387(98.44)	790(97.41)	0.0287	
	Yes (♂)	101(1.56)	21(2.59)		1.71(1.05, 2.77)*
Folacin intake duration	No	1453(22.71)	231(28.62)	0.0016	
	≥3 months before pregnancy	2372(37.07)	267(33.09)		0.70(0.58, 0.85)*
	1–2 months before pregnancy	1091(17.05)	125(15.49)		0.71(0.56, 0.90)*
	After pregnancy	1482(23.16)	184(22.80)		0.78(0.63, 0.96)*
Folacin Regular intake	Yes	4637(93.77)	534(92.55)	0.2213	
	No	308(6.23)	43(7.45)		1.24(0.88, 1.74)
Work or life pressure (♀)	1	5034(79.73)	620(78.58)	0.0189	
	2	759(12.02)	84(1.65)		0.91(0.71, 1.16)
	3	479(7.59)	80(1.14)		1.37(1.06, 1.78)*
	4	38(0.60)	3(0.38)		0.70(0.21, 2.27)
	5	4(0.06)	2(0.25)		5.44(0.91, 32.69)
Work or life exposure (♂)	1	4474(74.82)	559(74.93)	0.0060	
	2	805(13.46)	80(1.72)		0.80(0.62, 1.02)
	3	631(1.55)	89(11.93)		1.13(0.88, 1.45)
	4	61(1.02)	17(2.28)		2.41(1.37, 4.23)*
	5	9(0.15)	1(0.13)		0.97(0.12, 7.8)
Tense relationship with others(♀)	1	5691(9.43)	710(89.99)	0.0169	
	2	483(7.68)	55(6.97)		0.90(0.67, 1.21)
	3	117(1.86)	22(2.79)		1.58(0.99, 2.53)
	4	2(0.03)	2(0.25)		7.63(1.07, 54.25)*
Tense relationship with others (♂)	1	5341(89.51)	657(88.43)	0.1909	
	2	475(7.96)	61(8.21)		1.06(0.80, 1.40)
	3	145(2.43)	23(3.10)		1.29(0.82, 2.03)
	4	6(0.10)	2(0.27)		2.57(0.52, 12.72)
Economic pressure (♀)	1	4985(79.22)	620(78.68)	0.2394	
	2	714(11.35)	79(1.03)		0.89(0.70, 1.15)
	3	550(8.74)	82(1.41)		1.23(0.96, 1.58)
	4	36(0.57)	6(0.76)		1.34(0.55, 3.24)
	5	8(0.13)	1(0.13)		1.12(0.14, 9.08)
Economic pressure (♂)	1	4455(74.74)	550(74.02)	0.0736	
	2	776(13.02)	80(1.77)		0.86(0.67, 1.10)
	3	647(1.85)	97(13.06)		1.24(0.98, 1.58)
	4	61(1.02)	11(1.48)		1.53(0.79, 2.95)
	5	22(0.37)	5(0.67)		1.87(0.68, 5.14)
Ready for Pregnancy (♀)	No	274(87.54)	39(12.46)	0.4182	
	Yes	6048(88.97)	750(11.03)		0.87(0.61, 1.22)
Ready for Pregnancy (♂)	No	223(3.72)	33(4.44)	0.2986	
	Yes	5765(96.28)	710(95.56)		0.82(0.56, 1.19)

*For *P* < 0.05.

Parental exposed to working or life stress was associated with stillbirth, but the relationship was not linear. Economic stress and got ready for pregnancy were not statistically significant related with stillbirth (Table 3).

We found that folacin intake was a protective factor of stillbirth in our study (P = 0.0016), the OR was similar for those who take folacin at least 3 months before pregnancy (OR = 0.70 (95%CI 0.58, 0.85)) or 1–2 months before pregnancy (OR = 0.71 (95%CI 0.56, 0.90)*), and slightly higher in those who take folacin after getting pregnant (OR = 0.78 (95%CI 0.63, 0.96)). And as long as the mothers take folacin, it’s not significantly different between those who take regularly and who not (P = 0.2213) (Table 3).

To illustrate the relationship between vegetables consumption and stillbirth, we stratified the data by folacin intake. For both who take folacin and who don’t, lack of vegetables seemed to be a risk factor, with OR of 3.60(95% CI 1.04, 12.38) and 2.04(95% CI 0.94, 4.43) respectively (Table 4).

**Table 4:** Relationship between lack of vegetables and stillbirth stratified by folacin intake.

Folacin intake	Lack of vegetables	Live birth	Stillbirth	*P*	OR(95% *CI*)
No	No	1378(99.49)	219(98.21)	0.0425	
	Yes	7(0.51)	4(1.79)		3.60(1.04, 12.38)*
Yes	No	4790(99.3)	553(98.57)	0.0532	
	Yes	34(0.70)	8(1.43)		2.04(0.94, 4.43)*

*For P < 0.05.

Multi-level logistic model of risk factors of maternal and paternal were analyzed independently. TOL of variables in both models were all greater than 0.1, and VIF were all less than 10, so that we assume no collinearity among variables in the two models respectively. Overall tests of the two models were statistically significant, with both P < 0.001. Tests of Random parameter was also significant, with P = 0.03045 < 0.05, which mean that multi-level models should be used.

Multi-level logistic regression showed that maternal exposure to pesticide (OR (95%CI 1.06, 3.39)), maternal hypertension (OR = 1.58 (95%CI 1.07, 2.34)), lack or loss of appetite for vegetables (OR = 1.99 (95%CI 1.00, 3.93)), maternal pressure (compared with no pressure, OR of a little pressure was 1.34 (95% CI 1.02, 1.76)), paternal smoking (OR = 1.22 (95% CI 1.02, 1.46)), paternal exposing to Organic solvents (OR = 1.64 (95% CI 1.01, 2.69)) were independent risk factors of stillbirth. Folacin intake 3 months before pregnancy (OR = 0.72 (95% CI 0.59, 0.89)), folacin intake 1-2 months before pregnancy (OR = 0.71 (95% CI 0.55, 0.92)), folacin intake after pregnancy (OR = 0.81 (95% CI 0.65, 1.02) for) were protect factors of stillbirth compared with no folacin intake (Table 5).

**Table 5:** Multi-level logistic regression of parental risk factors of stillbirth.

Variables	Estimate	Se.	*P*-Value	OR(95%*CI*)
**Maternal**				
intercept	−1.7154	0.2387	<0.0001	0.18(0.11, 0.29)
Folacin 1-0	−0.3217	0.1054	0.0031	0.72(0.59, 0.89)*
Folacin 2-0	−0.3429	0.1289	0.0095	0.71(0.55, 0.92)*
Folacin 3-0	−0.2081	0.1138	0.0712	0.81(0.65, 1.02)
Pesticide	0.6414	0.2952	0.0298	1.90(1.06, 3.39)*
Blood high pressure	0.458	0.2008	0.0226	1.58(1.07, 2.34)*
Lack of vegetables	0.6857	0.349	0.0495	1.99(1.00, 3.93)*
Maternal passive smoking	0.1781	0.1132	0.1155	1.19(0.96, 1.49)
Pressure 1-0	−0.04218	0.1287	0.7441	0.96(0.74, 1.24)
Pressure 2-0	0.2924	0.1377	0.0374	1.34(1.02, 1.76)*
Pressure 3-0	−0.2828	0.6121	0.6455	0.75(0.22, 2.56)
Pressure 4-0	0.8882	1.1239	0.4321	2.43(0.26, 22.88)
Age	−0.00624	0.0087	0.4708	0.99(0.98, 1.01)
Random parameter	0.06078	0.0316		
**Paternal**				
intercept	−1.9609	0.2423	<0.0001	0.14(0.09, 0.23)
smoking	0.2000	0.0902	0.0265	1.22(1.02, 1.46)*
drinking	0.0607	0.08367	0.4683	1.06(0.90, 1.25)
New decoration	0.4971	0.2510	0.0477	1.64(1.01, 2.69)*
age	−0.0062	0.0084	0.4583	0.99(0.98, 1.01)
Random parameter	0.0690	0.03619		

*For P < 0.05.

## Discussion

We found that stillbirth rate was much higher in the west, almost twice of the average rate. Due to low economic development level and sparse population, the maternal and child healthcare services provided in western areas were not as good as east. Some researches showed that the management rate, physical examination rate at early pregnancy, the visiting rate after delivery were all lower in western areas^33^. How to implement effective maternal and child health management for western areas are of vital important now.

Maternal exposure to pesticide was identified as an independent risk factor of stillbirth, which is consistent with previous studies^34–36^. Pesticide exposure are more common with farmers with lower education level. Due to limited health literacy, they know little about the harm of pesticide and protection methods. To protect people from the harm of pesticide, regular health education on how to apply pesticide safely should be provided, and specialized teams should be organized to help with the pesticide spraying^37,38^.

Maternal high blood pressure before pregnancy was also associated with stillbirth. According to previous studies, 4–7% of stillbirth happened due to high blood pressure during pregnancy, and high blood pressure before pregnancy which was not ideally control was an important cause of high blood pressure during pregnancy^39–42^. Reinforce the management of maternal blood pressure, and provide adequate treatment for those with high blood pressure before pregnancy are very important to reduce the stillbirth rate.

Folacin intake was proved to be protective factor of stillbirth, regardless of when the woman started the intake. And for those who rarely or don’t eat vegetables, the protective effect was larger. Previous studies showed that the rate of folacin intake among women in rural China is rising these years, which is a good phenomenon. It is important for basic public health services departments to increase the rate of folacin intake in a standardized way^18^.

Stress in daily life or work is also a risk factor of stillbirth, which may be a bigger problem for women in business. But it is worthwhile to notice that the stillbirth rate showed no statistically significance between those who got ready for this baby and those who not.

For paternal risk factors, paternal smoking, paternal drinking, and paternal exposed to new house decoration were all associated with stillbirth after adjustment of paternal age. In rural China, male smoking and drinking are still severe public health problems that need to be addressed^43,44^. New decorated house may be associated with higher dosage level of formaldehyde or organic solvent, which were reported to be risk factors of stillbirth^45^.

Limitations of our studies include that many of risk factors collected by our study were binary data, which made the analysis of dose-response relationship impossible, the exposing status such as smoking, drinking or pesticide were self-reported, which may not be very accurate. Also, the participants were not randomly selected, which may also cause some bias of our study.

### Ethical approval

The study was approved by the institutional research review board at the National Health and Family Planning Commission and National Research Institute for Family Planning. Informed consents were obtained from all participants or their legal representatives. All research was performed in accordance with relevant guidelines.

## Data Availability

The data generated cannot be made publicly available according to the Chinese law of personal data protection and also our project data management rules. However, data inquires or further suggestions for analyses can be made to the corresponding author.
